# Association between Low Free Triiodothyronine Levels and Poor Prognosis in Patients with Acute ST-Elevation Myocardial Infarction

**DOI:** 10.1155/2018/9803851

**Published:** 2018-04-16

**Authors:** Yuanbin Song, Jiabei Li, Shizhu Bian, Zhexue Qin, Yaoming Song, Jun Jin, Xiaohui Zhao, Mingbao Song, Jianfei Chen, Lan Huang

**Affiliations:** ^1^Department of Cardiology, Xinqiao Hospital, Third Military Medical University, Chongqing 400037, China; ^2^PLA Institute of Cardiovascular Disease, Chongqing 400037, China

## Abstract

**Background:**

Low free triiodothyronine (fT3) levels are generally associated with poor prognosis in patients with heart diseases, but this is controversial and there is a lack of data about ST-elevation myocardial infarction (STEMI) in Chinese patients.

**Objective:**

To assess the association between fT3 levels and the prognosis of patients with STEMI.

**Methods:**

This was a prospective observational study of 699 consecutive patients with STEMI treated at the Xinqiao Hospital between January 1, 2013, and December 31, 2014. The patients were divided into the low fT3 (fT3 < 3.1 pmol/L; *n* = 179, 27.5%) and normal fT3 (fT3 ≥ 3.1 pmol/L; *n* = 473, 72.5%) groups according to fT3 levels at admission. Patients were followed up at 1, 3, 6, and 12 months for all-cause death and major adverse cardiac events (MACE).

**Results:**

During the 1-year follow-up, there were 70 all-cause deaths (39.1%) in the low fT3 group and 40 (8.5%) in the normal fT3 group (*P* < 0.001). MACE occurred in 105 patients (58.7%) in the low fT3 group and 74 (15.6%) in the normal fT3 group (*P* < 0.001). Multivariate Cox proportional hazards regression analysis indicated that fT3 levels were independently associated with 30-day and 1-year all-cause death [30-day: hazard ratio (HR) = 0.702, 95% confidence interval (95% CI): 0.501–0.983, *P* = 0.04; 1-year: HR = 0.557, 95% CI: 0.411–0.755, *P* < 0.001] and MACE (30-day: HR = 0.719, 95% CI: 0.528–0.979, *P* = 0.036; 1-year: HR = 0.557, 95% CI: 0.445–0.698, *P* < 0.001).

**Conclusion:**

Low fT3 levels were strongly associated with poor prognosis in patients with STEMI. Measurement of fT3 levels may be a valuable and simple way to identify high-risk STEMI patients.

## 1. Introduction

Thyroid hormones (THs) metabolism is altered in severe illnesses and is characterized by low serum free triiodothyronine (fT3) levels and normal-to-low free thyroxine (fT4) and thyroid-stimulating hormone (TSH) levels [1]. This altered pattern is known as the low-T3 syndrome or euthyroid sick syndrome [1]. The decline of fT3 levels may be caused by decreased conversion of the prohormone T4 into T3 and by increased T3 catabolism [2–4]. Inflammation, hypoxia, and oxidative stress have been found to be involved in the downregulation of T3 levels by modulating the activity of deiodinase [5–7].

THs have profound effects on the cardiovascular system. As the main biologically active hormone, fT3 plays a major role in increasing heart rate and cardiac contractility and decreasing systemic vascular resistance through genomic and nongenomic pathways [2]. Indeed, fT3 modulates the transcription of genes such as myosin heavy chain, phospholamban, sarcoplasmic reticulum Ca^2+^-ATPase, and Na^+^/Ca^2+^ exchanger, which are all proteins involved in the normal function of the heart [8].

Inflammation, hypoxia, and oxidative stress are conditions that are often found in patients with heart diseases [9–11]. Low fT3 levels are associated with higher right atrial, pulmonary artery, and pulmonary capillary wedge pressures and with lower ejection fraction and cardiac index [12]. Accordingly, the low-T3 syndrome is a common finding and a predictor of poor short- and long-term prognosis in patients with heart failure and acute ST-elevation myocardial infarction (STEMI) [13–19]. Nevertheless, the association between low fT3 levels and poor cardiovascular outcomes in patients with heart diseases remains controversial [20–22]. In a literature review, Lamprou et al. [23] suggested that although fT3 levels show promising prognostic value in acute coronary syndromes, the association remains uncertain. Therefore, the clinical value of fT3 in patients with cardiovascular diseases remains to be determined using well-designed prospective studies [23].

Taken together, the association between fT3 levels and heart diseases is controversial, and there is a lack of data specific to STEMI. In addition, thyroid function and the incidence/morbidity of heart diseases vary with ethnicity [24–26], and few studies examined the relationship between fT3 levels and cardiac prognosis in Chinese patients. Therefore, the aim of this prospective observational study was to investigate the association between fT3 levels and the prognosis of Chinese patients with STEMI. The results could provide a new biomarker for the prognosis of patients with STEMI. Measurement of fT3 levels could be a valuable and simple way to identify high-risk STEMI patients and improve their management.

## 2. Materials and Methods

### 2.1. Study Design and Patients

This was a prospective observational study of 699 consecutive patients diagnosed with STEMI and treated at the Department of Cardiology, Xinqiao Hospital, from January 1, 2013, to December 31, 2014.

The inclusion criterion was a diagnosis of STEMI according to the 2009 ACCF/AHA guidelines for the management of STEMI: characteristic chest pain, ST-segment elevation or new left bundle branch block, and elevated troponin I (TnI) levels [27]. The exclusion criteria were (1) history of thyroid disease, (2) overt hypothyroidism or hyperthyroidism, (3) no baseline THs data, (4) amiodarone use within one month, or (5) refusal to participate.

The study was approved by the Ethics Committee of Xinqiao Hospital, Third Military Medical University (Chongqing, China). Written informed consent was obtained from the patients or a legal representative.

### 2.2. Data Collection

Demographic and clinical data (age, sex, smoking, history of myocardial infarction, diabetes mellitus, and hypertension) were obtained from all patients. All patients underwent electrocardiogram (ECG) examination, blood pressure and heart rate measurements, and physical examination after admission.

### 2.3. Biochemistry

Fasting blood samples were collected within the first 24 h after admission. The serum levels of THs (including fT3, fT4, and TSH) were measured using electrochemiluminescence immunoassays (Cobas E601 immunology analyzer, Roche, Mannheim, Germany). Cardiac TnI and brain natriuretic peptide (BNP) were measured using a Triage MeterPro system (Alere, San Diego, CA, USA) and immunofluorescence assay kits (Shijiazhuang, Hebei, China). The levels of peak cardiac TnI (measured over time after admission) and the levels of BNP at admission were compared between the two groups.

### 2.4. Grouping

At our hospital, the reference values for fT3, fT4, and TSH are 3.1–6.8 pmol/L, 11.0–22.0 pmol/L, and 0.27–4.2 mIU/L, respectively. The patients were divided into the low fT3 group (fT3 < 3.1 pmol/L) and the normal fT3 group (fT3 ≥ 3.1 pmol/L) according to fT3 levels at admission [28].

### 2.5. Follow-Up

Follow-up started on the day of admission and was performed at 1, 3, 6, and 12 months after discharge by outpatient visits (preferably) or phone.

### 2.6. Outcomes

The primary outcome was all-cause death (death from any natural cause). The secondary outcome was the incidence of major adverse cardiac event (MACE), defined as any of the following: cardiac-related death, rehospitalization for heart failure, or nonfatal myocardial infarction. Cardiac-related death was defined as the documentation of significant arrhythmia or cardiac arrest, death from congestive heart failure, or myocardial infarction in the absence of any other precipitating factors [28]. In case of out-of-hospital death, sudden unexpected death was regarded as cardiac-related death, unless proven otherwise by autopsy. Deaths caused by accidents were excluded (the follow-up was censored at the time of death) [28]. The patients lost to follow-up were censored at the last contact.

### 2.7. Statistical Analysis

The distribution of continuous data was tested with the Kolmogorov-Smirnov test. Continuous variables were presented as mean ± standard deviation (normally distributed) or median (25th–75th percentile) (non-normally distributed) and analyzed using Student's *t*-test (normally distributed) or Mann–Whitney *U* test (non-normally distributed). Categorical variables were presented as counts and percentages and analyzed using Pearson's chi-square or Fisher's exact test, as appropriate. The Cox proportional hazard regression model was used to determine the univariate and multivariate hazard ratios (HR) with 95% confidence intervals (CI) for the primary and secondary outcomes. Continuous variables [age, fT3, fT4, TSH, TnI, BNP, total cholesterol (TC), low-density lipoprotein cholesterol (LDL-C), high-density lipoprotein cholesterol (HDL-C), triglycerides (TG), serum creatinine, systolic blood pressure (SBP), diastolic blood pressure (DBP), heart rate, and left ventricular ejection fraction (LVEF)] and categorical variables (sex, history of hypertension, diabetes, smoking, infarction site, Killip class, and reperfusion therapy) were entered into the model and analyzed by forward stepwise regression. The Kaplan-Meier method and the log-rank test were used to analyze all-cause death and MACE occurrence during follow-up. All statistical analyses were carried out using SPSS 13.0 for Windows (SPSS Inc., Chicago, USA). Two-sided* P* values < 0.05 were considered statistically significant. 

## 3. Results

### 3.1. Patients

Forty-seven patients were excluded: history of thyroid diseases (*n* = 7), overt hypothyroidism (*n* = 8), overt hyperthyroidism (*n* = 9), amiodarone use within one month (*n* = 2), no baseline THs data (*n* = 5; THs were not measured because of death within 5 h after admission), and refusal to participate (*n* = 16). The remaining 652 patients were enrolled in this study.

### 3.2. Baseline Characteristics

Among the 652 patients, 179 (27.5%) were in the low fT3 group and 473 (72.5%) were in the normal fT3 group. The baseline characteristics are shown in Table 1. Compared with the normal fT3 group, patients in the low fT3 group were older (*P* < 0.001), had lower proportions of male sex (*P* < 0.001 but *P* = 0.071 after adjustment for age) and current smoker (*P* < 0.001 but *P* = 0.568 after adjustment for age and sex), had a higher proportion of high Killip class (Killip class > I) (62.6% versus 27.5%, *P* < 0.001), had higher heart rates at admission (*P* < 0.001), had higher serum creatinine levels (unadjusted and adjusted *P* < 0.001), and had lower SBP, DBP, fT3, fT4, TSH, TC, LDL-C, TG, and hemoglobin (all unadjusted and adjusted *P* < 0.05). There were no significant differences in hypertension, diabetes, history of myocardial infarction, and HDL-C levels between the two groups (all *P* > 0.05). Among all patients, 90 (50.3%) in the low fT3 group and 342 (72.3%) in the normal fT3 group received reperfusion therapy using primary percutaneous coronary intervention (PCI) or thrombolytic therapy (unadjusted and adjusted *P* < 0.01).

### 3.3. BNP, TnI, and LVEF

The peak levels of cardiac TnI were significantly higher in the low fT3 group than in the normal fT3 group (*P* = 0.017) (Table 1). The levels of BNP at admission in the low fT3 group were significantly higher than in the normal fT3 group (*P* < 0.001) (Table 1). The LVEF was significantly lower in the low fT3 group than in the normal fT3 group (*P* = 0.001) (Table 1).

### 3.4. Association between fT3 and All-Cause Death

**Figure 1:**
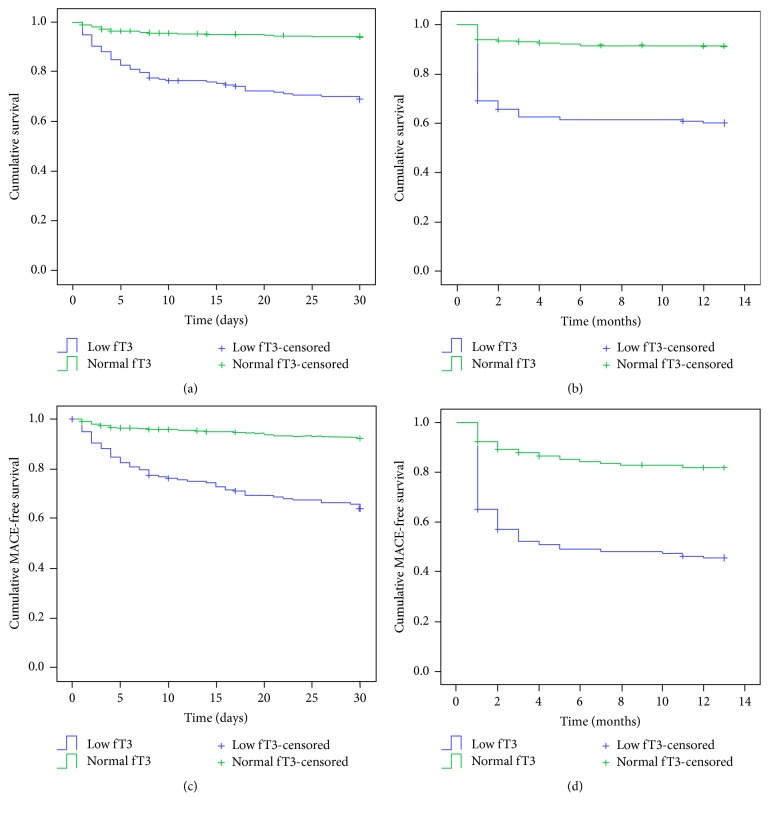
Kaplan-Meier survival curves for (a) 30-day mortality, (b) 1-year mortality, (c) 30-day major adverse cardiac events (MACE), and (d) 1-year MACE in patients in the low and normal fT3 groups. The log-rank tests showed that all four parameters (mortality and MACE occurrence) were higher in the low fT3 group than in the normal fT3 group (all *P* < 0.001). After adjustment for age and sex, all associations remained significant, except for 30-day and 1-year mortality and 30-day MACE in female >75 years of age (all *P* > 0.05); only the difference in 1-year MACE remained significant (*P* = 0.019).

The mean follow-up was 10.6 ± 4.7 months. A total of 31 patients were lost to follow-up: 9 (5.0%) in the low-fT3 group and 22 (4.7%) in the normal-fT3 group. During the first 30 days, there were 55 all-cause deaths (30.7%) in the low fT3 group and 27 (5.7%) in the normal fT3 group (*P* < 0.001) (Figure 1(a)). During the 1-year follow-up, 70 patients (39.1%) in the low fT3 group died, compared with 40 (8.5%) in the normal fT3 group (*P* < 0.001) (Figure 1(b)). The multivariate Cox proportional hazard regression analysis indicated that fT3 levels were independently associated with 30-day all-cause death (for each 1 pmol/L increase, HR: 0.702, 95% CI: 0.501–0.983, *P* = 0.040) and 1-year all-cause death (for each 1 pmol/L increase, HR: 0.557, 95% CI: 0.411–0.755, *P* < 0.001) (Table 2).

### 3.5. Association between fT3 and MACE

During the first 30 days, there were 63 MACE (35.2%) in the low fT3 group, compared with 36 (7.6%) in the normal fT3 group (*P* < 0.001) (Figure 1(c)). During the 1-year follow-up period, 95 MACE (53.1%) occurred in patients with low fT3 levels, compared with 84 (17.8%) in the normal fT3 group (*P* < 0.001) (Figure 1(d)). Multivariate Cox hazard regression analysis suggested that fT3 was independently associated with 30-day MACE (for each 1 pmol/L increase, HR: 0.719, 95% CI: 0.528–0.979, *P* = 0.036) and 1-year MACE (for each 1 pmol/L increase, HR: 0.557, 95% CI: 0.445–0.698, *P* < 0.001) (Table 3).

### 3.6. Subgroup Analyses

fT3 levels were significantly associated with 1-year all-cause death and MACE in patients ≥75 and <75 years of age, male and female sex, with and without diabetes, with and without history of hypertension, anterior wall infarction and others, and Killip class I and others (all unadjusted *P* < 0.001). After adjustment for age and sex, all associations remained significant, except 30-day and 1-year mortality and 30-day MACE in female >75 years of age (all *P* > 0.05); only the difference in 1-year MACE remained significant (*P* = 0.019). In patients who received primary PCI, fT3 levels were significantly associated with 1-year mortality and MACE (all *P* < 0.001). In patients who received thrombolytic therapy, fT3 levels were significantly associated with 1-year mortality (*P* = 0.020), but not with 1-year MACE (*P* = 0.076).

## 4. Discussion

Low fT3 levels are generally associated with poor prognosis in patients with heart diseases [13–18], but this is controversial [20, 21] and there is a lack of data about Chinese patients with STEMI. Therefore, this study aimed to assess the association between fT3 levels and prognosis of Chinese patients with STEMI. The results showed that low fT3 levels are a strong predictor of poor prognosis in patients with STEMI. Measurement of fT3 levels may be a valuable and simple way to identify high-risk STEMI patients. These results provide useful insights into the management of Chinese patients with STEMI.

Low T3 levels are frequently observed in serious illnesses of nonthyroidal origin [1]. A systematic review and meta-analysis recently showed that the prevalence of the low T3 syndrome is high in heart failure (24.5%), myocardial infarction (18.9%), and acute coronary syndrome (17.1%) [29]. The present study revealed a high rate (27.5%) of low fT3 levels among patients with STEMI, similar to previous studies from Kazakhstan, China, and Italy [13, 14, 30]. In addition, patients in the low fT3 group had more serious myocardial injury, as assessed by peak cardiac TnI levels, and more severe cardiac dysfunction, as assessed by LVEF and BNP levels. These results are consistent with previous studies from China [14, 19] and suggest that fT3 levels are associated with the severity of STEMI.

The low-T3 syndrome is a common finding and a predictor of poor short- and long-term prognosis in patients with heart failure and acute ST-elevation myocardial infarction (STEMI) from Kazakhstan, China, the United States of America, Japan, and Turkey [13–18]. A prospective study by Özcan et al. [13] in 457 STEMI patients from Kazakhstan found that serum fT3 levels had a trend toward associations with in-hospital and long-term MACE in univariate analyses, but not in multivariate analyses. This previous study only enrolled 30 patients with low fT3 levels and the rates of MACE were analyzed by logistic regression model, but this analysis does not consider the time-event factor [13]. A retrospective study by Zhang et al. [14] in 501 Chinese STEMI patients found that the 30-day and 1-year mortality and incidence of MACE in patients with low fT3 level were higher than in patients with normal fT3 levels. Their multivariate model showed that low fT3 levels were independently associated with short- and long-term death and MACE [14]. Rothberger et al. (United States) [15] and Okayama et al. (Japan) [16] suggested that fT3 levels could be used to stratify patients with heart failure at admission according to their risk. Kozdag et al. (Turkey) [17] showed that fT3 levels were associated with the prognosis of MACE in patients with chronic heart failure. Mitchell et al. [18] showed that TH levels were associated with the outcomes in American patients with severe heart failure. Wang et al. [19] showed that low fT3 levels were associated with high levels of markers of myocardial damage and with low LVEF after STEMI in Chinese patients.

Nevertheless, the association between low fT3 levels and poor cardiovascular outcomes in patients with heart diseases remains controversial [20, 21]. Indeed, Frey et al. [20] showed that the TH levels were not associated with the prognosis of heart failure. Perez et al. [21] showed that TH levels were not associated with the prognosis of heart failure with decreased ejection fraction. A prospective study conducted by Friberg et al. [22] in 331 patients with AMI suggested that T3 was not a significant predictor of 30-day and 1-year death, but they showed that mortality was nevertheless high among patients with the most pronounced TH depression. In a literature review, Lamprou et al. [23] suggested that although fT3 levels show promising prognostic value in acute coronary syndromes, additional studies are necessary to determine their exact contribution. Therefore, the clinical value of fT3 in patients with cardiovascular diseases remains to be determined using well-designed prospective studies [23].

In the present study of Chinese patients with STEMI, the Kaplan-Meier analyses showed that the mortality rates and occurrence of MACE in the low fT3 group were significantly higher than in the normal fT3 group, confirmed by the multivariate Cox proportional hazard regression analyses. In a recent systematic review and meta-analysis, the low-T3 syndrome was a significant predictor of all-cause death and MACE, supporting the present study [29]. Lymvaios et al. [31] showed that changes in T3 levels after AMI were correlated with early and late recovery of cardiac function and that 6-month T3 levels were an independent predictor of late functional recovery. Unfortunately, in the present study, fT3 levels were measured only at admission.

In China, because of health insurance coverage, not all patients with STEMI have the opportunity to receive reperfusion therapy in the acute phase [32], resulting in reperfusion therapy rates lower than in western countries [33, 34] and higher mortality rates. In the present study, 66.3% of the patients received primary PCI or thrombolytic therapy. Accordingly, the results showed that the 1-year mortality rates for patients who received and did not receive PCI were 6.9% (30/432) and 36.4% (80/220), respectively. The subgroup analyses revealed that fT3 levels were associated with mortality and MACE in all patients, that is, those who received primary PCI and those who did not receive reperfusion therapy in the acute phase. On the other hand, fT3 levels were associated with mortality in patients who received thrombolytic therapy, but not with MACE. Pavlou et al. [35] observed similar prognostic value of fT3 in patients with or without thrombolytic therapy. Additional studies are necessary to examine the association between low fT3 levels and the efficacy of different treatment approaches for heart diseases.

Lazzeri et al. [30] found that, among patients <75 years of age, those with low fT3 exhibited a significantly lower survival rate, while among patients ≥75 years of age, no differences were observed in long-term survival between patients with low and normal fT3 levels [30]. Wang et al. [19] found no association between fT3 levels and mortality according to age. Consistent with this previous study [30], we found that the 1-year mortality rate of patients <75 years of age was significantly higher in the low fT3 group compared with the normal fT3 group (*P* < 0.001). On the other hand and in disagreement with this previous study [30], we found that the 1-year mortality rate of patients ≥75 years of age was also significantly higher in the low fT3 group compared with the normal fT3 group (*P* < 0.001).

Inflammatory cytokines and oxidative stress encountered in several illnesses (including STEMI) suppress the production of thyrotropin-releasing hormone in the hypothalamus, decreasing the T3 levels [36, 37]. Interleukin-6 (IL-6) (an inflammatory cytokine) correlates negatively with fT3 levels [38]. Tumor necrosis factor-*α* (TNF-*α*) and interferon-*γ* (IFN-*γ*) lead to decreased fT3 levels [39, 40]. Therefore, inflammation probably contributes to low T3 levels.

Animal studies showed that giving THs after AMI improved cardiac function and remodeling [41–43]. It has been speculated that the low T3 state may result in a hypothyroid-like syndrome that leads to the deterioration of AMI. THs have antiapoptosis, mitochondrial protection, cell growth and differentiation, induction of myocardial hypertrophy, neoangiogenesis, and antifibrosis activities, which are considered to be cardioprotective [2, 44]. Although the beneficial effects of TH treatment after AMI have been shown in animal studies, whether the low T3 state is only a biological risk factor or a direct causal factor contributing to the exacerbation of STEMI remains to be determined. To answer this question, the demonstration of beneficial effects on cardiovascular endpoints of long-term T3 replacement therapy in cardiac patients with low-T3 syndrome is needed. Animal studies suggest that supplementing THs after cardiac ischemia could improve the cardiac indexes and survival [11, 45, 46], but these results will have to be confirmed in humans.

The thyroid gland mainly secretes two hormones [T3 and thyroxine (T4)] and T3 is considered to be the biologically active one. More than 80% of T3 is derived from the peripheral conversion of prohormone T4 by deiodination [2]. Two deiodinase enzymes [type I iodothyronine deiodinase (DIO1) and type II iodothyronine deiodinase (DIO2)] lead to T4 deiodination and T3 production [2]. The third deiodinase [thyroxine 5-deiodinase (DIO3)] catabolizes both T3 and T4 to inactive products, leading to the termination of TH action [2]. Previous studies revealed that the decline of T3 levels after AMI may be caused by increased DIO3 activity and reduced activities of DIO1 and DIO2 [3, 4]. In the present study, fT4 levels were independently associated with 1-year all-cause mortality and MACE occurrence. Since fT4 is biologically inactive, this association could be due to the activities of DIO1, DIO2, and DIO3, but additional studies are needed to determine the exact mechanisms involved in the changes in THs after AMI. Nevertheless, animal studies suggested that Akt, ERK, and HSP70 signaling pathways are involved in the effects of THs on postischemia cardiac performance [11, 46]. In addition, THs decrease cardiomyocyte apoptosis [45]. Decreased TH receptor *α*1 may also be involved in the low fT3 syndrome and in the effects of THs on cardiac repair after ischemia [46–49]. These results from animal models could provide some hints toward the mechanisms in humans.

The main strengths of the present study are the large sample size and the relatively low rate of loss to follow-up. Nevertheless, there are some limitations. First, THs were not examined at various time points and the changes of THs over time after AMI are unknown. The optimal timing of TH measurement after AMI is still largely unknown. Secondly, not all patients received reperfusion therapy in the acute phase. Thirdly, the follow-up was relatively short. Finally, all patients were Chinese, limiting the generalizability of the results. Additional studies are necessary to address these issues.

## 5. Conclusion

Low fT3 levels are strongly associated with poor prognosis in patients with STEMI. Measurement of fT3 levels may be a valuable and simple way to identify high-risk STEMI patients. These results provide useful insights into the management of Chinese patients with STEMI. Prognostic algorithms and scoring systems that include fT3 could be explored.

## Figures and Tables

**Table 1:** Baseline characteristics of the patients.

	Low fT3 (*n* = 179)	Normal fT3 (*n* = 473)	*P*	Adjusted *P*^*∗*^
Age (years)	68 (59–76)	61 (50–71)	<0.001	<0.001^¶^
Male gender, *n* (%)	113 (63.1)	390 (82.5)	<0.001	0.071^‡^
Current smoker, *n* (%)	63 (35.2)	253 (53.5)	<0.001	0.568
History of hypertension, *n* (%)	88 (49.2)	206 (43.6)	0.199	0.939
Diabetes, *n* (%)	42 (23.5)	93 (19.7)	0.285	0.523
Previous MI, *n* (%)	6 (3.4)	15 (3.2)	0.907	0.884
SBP (mmHg)	112 (99–128)	119 (106–132)	0.003	<0.001
DBP (mmHg)	70 (61–79)	71 (64–82)	0.038	0.050
Heart rate (bpm)	86 (74–102)	78 (68–90)	<0.001	<0.001
Killip class on admission, *n* (%)			<0.001	<0.001
I	67 (37.4)	343 (72.5)		
II	38 (21.2)	78 (16.5)		
III	32 (17.9)	17 (3.6)		
IV	42 (23.5)	35 (7.4)		
LVEF (%)	58 (48–61)	59 (55–62)	0.001	0.001
fT3 (pmol/L)	2.5 (2.3–2.8)	3.8 (3.4–4.2)	<0.001	<0.001
fT4 (pmol/L)	14.8 (13–17.1)	15.2 (13.6–17.1)	0.018	0.001
TSH (mIU/L)	1.17 (0.65–2.05)	1.47 (0.79–2.43)	0.007	0.003
Serum creatinine (*μ*mol/L)	80.9 (64.1–112.9)	73.5 (62.8–88.9)	<0.001	<0.001
BNP (pg/ml)	745.5 (389.5–1585.0)	257.0 (90.0–574.3)	<0.001	<0.001
TnI (ng/ml)	11.5 (4.6–22.4)	8.0 (2.4–20.1)	0.017	0.050
TC (mmol/L)	4.08 (3.26–4.71)	4.32 (3.63–5.02)	0.003	0.005
LDL-C (mmol/L)	2.40 (1.90–3.04)	2.64 (2.15–3.16)	0.003	0.006
TG (mmol/L)	1.22 (0.96–1.66)	1.48 (1.10–2.09)	<0.001	<0.001
HDL-C (mmol/L)	0.97 (0.81–1.17)	0.94 (0.80–1.13)	0.424	0.453
Hemoglobin (g/L)	121 (108–134)	134 (122–145)	<0.001	<0.001
Reperfusion therapy, *n* (%)	90 (50.3)	342 (72.3)	<0.001	0.001
Thrombolytic therapy, *n* (%)	24 (13.4)	68 (14.4)	0.751	0.497
Primary PCI, *n* (%)	66 (36.9)	274 (57.9)	<0.001	<0.001
Aspirin, *n* (%)	173 (96.65)	466 (98.52)	0.204	0.438
Clopidogrel/Ticagrelor, *n* (%)	175 (97.77)	468 (98.94)	0.268	0.588
*β*-receptor blocker, *n* (%)	140 (78.21)	388 (82.03)	0.268	0.659
Statin, *n* (%)	165 (92.18)	449 (94.93)	0.181	0.257
ACEI/ARB, *n* (%)	144 (80.45)	407 (86.05)	0.078	0.134

^*∗*^Adjusted for age and sex. ^¶^Adjusted for sex. ^‡^Adjusted for age. MI: myocardial infarction; SBP: systolic blood pressure; DBP: diastolic blood pressure; LVEF: left ventricular ejection fraction; fT3: free triiodothyronine; fT4: free thyroxine; TSH: thyroid-stimulating hormone; BNP: brain natriuretic peptide; TnI: troponin I; TC: total cholesterol; LDL-C: low-density lipoprotein cholesterol; TG: triglycerides; HDL-C: high-density lipoprotein cholesterol; PCI: percutaneous coronary intervention; ACEI: angiotensin converting enzyme inhibitor; ARB: angiotensin II receptor blocker.

**Table 2:** Multivariate Cox proportional hazard regression analysis of predictors of 30-day and 1-year all-cause death.

Variables	Hazard ratio	95% CI	*P*
*30-day*			
Female sex	2.195	1.296–3.718	0.003
Killip class	1.847	1.472–2.316	<0.001
Ejection fraction	0.956	0.932–0.981	0.001
fT3	0.702	0.501–0.983	0.040
Serum creatinine	1.003	1.001–1.006	0.004
TnI	1.041	1.019–1.065	<0.001
Reperfusion	0.272	0.152–0.486	<0.001

*1-year*			
Female sex	1.793	1.129–2.848	0.013
Age	1.027	1.007–1.047	0.009
Heart rate	1.015	1.005–1.025	0.003
Killip class	1.466	1.209–1.779	<0.001
Ejection fraction	0.964	0.943–0.986	0.001
fT3	0.557	0.411–0.755	<0.001
fT4	1.096	1.027–1.169	0.006
TnI	1.029	1.010–1.050	0.003
HDL-C	0.444	0.204–0.968	0.041
Reperfusion	0.363	0.225–0.585	<0.001

fT3: free triiodothyronine; fT4: free thyroxine; TnI: troponin I; HDL-C: high-density lipoprotein cholesterol.

**Table 3:** Multivariate Cox proportional hazard regression analysis of predictors of 30-day and 1-year MACE.

Variables	Hazard ratio	95% CI	*P*
*30-day*			
Female sex	1.810	1.120–2.924	0.015
Heart rate	1.019	1.007–1.031	0.002
DBP	0.975	0.957–0.992	0.006
Killip class	1.449	1.177–1.785	<0.001
Ejection fraction	0.961	0.940–0.983	<0.001
fT3	0.719	0.528–0.979	0.036
Serum creatinine	1.004	1.002–1.004	<0.001
TnI	1.041	1.020–1.062	<0.001
Reperfusion	0.365	0.224–0.595	<0.001

*1-year*			
Killip class	1.338	1.156–1.549	<0.001
Ejection fraction	0.971	0.955–0.987	<0.001
fT3	0.557	0.445–0.698	<0.001
fT4	1.067	1.014–1.123	0.013
TSH	1.100	1.009–1.199	0.031
Reperfusion	0.497	0.358–0.692	<0.001

MACE: major adverse cardiac event; DBP: diastolic blood pressure; fT3: free triiodothyronine; fT4: free thyroxine; TnI: troponin I; TSH: thyroid-stimulating hormone.
